# Innate Immune Evasion of PRRSV nsp11 through Degradation of the HDAC2 by Its Endoribonuclease Activity

**DOI:** 10.3390/v16050678

**Published:** 2024-04-25

**Authors:** He Zhang, Jianxing Chen, Changqing Yu, Yu Pan, Wenjie Ma, Hao Feng, Jinxin Xie, Hongyan Chen, Yue Wang, Changyou Xia

**Affiliations:** 1 State Key Laboratory for Animal Disease Control and Prevention, Harbin Veterinary Research Institute, Chinese Academy of Agricultural Sciences, Harbin 150001, China; 2 School of Advanced Agricultural Sciences, Yibin Vocational and Technical College, Yibin 644000, China; 3 College of Veterinary medicine, Xinjiang Agricultural University, Urumqi 830000, China; xiejinxin198683@163.com

**Keywords:** PRRSV, HDAC2, nsp11, antagonize, endoribonuclease

## Abstract

Porcine reproductive and respiratory syndrome virus (PRRSV), a member of the *Arteriviridae* family, represents a persistent menace to the global pig industry, causing reproductive failure and respiratory disease in pigs. In this study, we delved into the role of histone deacetylases (HDAC2) during PRRSV infection. Our findings revealed that HDAC2 expression is downregulated upon PRRSV infection. Notably, suppressing HDAC2 activity through specific small interfering RNA led to an increase in virus production, whereas overexpressing HDAC2 effectively inhibited PRRSV replication by boosting the expression of IFN-regulated antiviral molecules. Furthermore, we identified the virus’s nonstructural protein 11 (nsp11) as a key player in reducing HDAC2 levels. Mutagenic analyses of PRRSV nsp11 revealed that its antagonistic effect on the antiviral activity of HDAC2 is dependent on its endonuclease activity. In summary, our research uncovered a novel immune evasion mechanism employed by PRRSV, providing crucial insights into the pathogenesis of this virus and guiding the development of innovative prevention strategies against PRRSV infection.

## 1. Introduction

Porcine reproductive and respiratory syndrome virus (PRRSV), belonging to the family *Arteriviridae*, is an enveloped, positive, single-stranded RNA virus [1,2,3]. As the causative agent of porcine reproductive and respiratory syndrome (PRRS), PRRSV is notorious for causing reproductive failures in sows and severe respiratory symptoms in pigs of all ages [4,5,6,7]. Since its emergence in the 1980s, PRRS has spread worldwide, posing a significant economic burden on the global swine industry [8,9]. The virus exhibits extensive genetic and antigenic diversity, leading to the emergence of numerous new strains and limiting the effectiveness of current vaccines. PRRSV infection is characterized by its ability to evade the host’s innate immune response, resulting in delayed protective antibody production and cell-mediated immune responses, which contributes to the complexity of PRRS prevention and control [10,11]. Therefore, a deeper and more comprehensive understanding of PRRSV antagonizing the host antiviral response is critical for developing novel therapeutic strategies to combat this virus.

The innate immune system of the host serves as the initial defense barrier against pathogenic microorganisms, providing a swift and nonspecific response to infections. However, PRRSV, through their prolonged confrontation with host cells, have evolved intricate immune evasion strategies to counter the host’s immune pathways and factors [12]. Accumulating evidence has demonstrated that several nonstructural and structural proteins, including the nsp1α [13,14], nsp1β [15], nsp2 [16], nsp4 [17], nsp11 [18], and N protein [19], play crucial roles in offsetting the host defense system through diverse mechanisms. For instance, nsp4 cleaves ZC3HAV1/ZAP and DCP1A, thereby inhibiting their anti-PRRSV activity [20,21], nsp3 induces the degradation of IFITM1, reducing its anti-PRRSV activity through the proteasome-dependent degradation pathway [22], and E protein degrades host restriction factor porcine CH25H via the ubiquitin–proteasome pathway [23] and degrades DDX10 through SQSTM1-dependent selective autophagy [24]. Therefore, screening for novel antiviral factors and identifying novel targets for the development of effective anti-PRRSV drugs is imperative. 

The histone deacetylases (HDACs) constitute a family of host enzymes responsible for catalyzing the deacetylation of acetylated proteins. Acetylation, a common posttranslational modification, holds crucial roles in regulating gene expression, cell cycle progression, signal transduction, and innate immune responses. This modification occurs across a wide range of nuclear and cytoplasmic proteins [25,26]. Conversely, deacetylation, a reversible process primarily controlled by HDACs, is essential in various cellular processes, including gene expression, cell cycle progression, signaling, and innate immune responses [27,28]. However, the impact of HDAC2 on PRRSV infection remains unclear.

In this study, we demonstrated that PRRSV infection could downregulate the endogenous expression of HDAC2 in host cells. Furthermore, ectopic expression of HDAC2 exhibited significant anti-PRRSV activity by promoting the expression of IFN-regulated antiviral molecules, whereas knockdown of HDAC2 enhanced PRRSV proliferation. Through screening PRRSV-encoded structural and nonstructural proteins, we discovered that the viral nonstructural protein 11 (nsp11) can reduce HDAC2 expression, dependent on its endoribonuclease activity. This downregulation of HDAC2 by nsp11 leads to enhanced PRRSV infection by blocking the antiviral effect of HDAC2. In summary, our study uncovers a novel strategy employed by PRRSV to counteract host antiviral innate immunity, providing a deeper understanding of the mechanisms underlying PRRSV infection.

## 2. Materials and Methods

### 2.1. Cell Culture and Viruses

The immortalized PAM cell line, kindly provided by Dr. Yan-dong Tang [29], was cultured in RPMI-1640 medium (Gibco, Waltham, MA, USA) supplemented with 10% fetal bovine serum (FBS, Gibco, Waltham, MA, USA). Marc-145 and HEK-293T cells were maintained in Dulbecco’s modified Eagle’s medium (DMEM, Gibco, Waltham, MA, USA) supplemented with 10% FBS. The HuN4 HP-PRRSV strain (GenBank no. EF635006) was propagated and titrated in Marc-145 cells.

### 2.2. Constructs and Antibodies

HDAC2 was amplified from PAMs’ cDNA using the primers listed in Table 1 and subcloned into the pCAGGS vector with a C-terminal HA tag. The recombinant pCAGGS plasmids encoding individual PRRSV viral proteins (nsp1α, nsp1β, nsp4, nsp5, nsp7, nsp9-11, ORF2α, ORF5, and ORF7) were fused with a Flag tag and were already available in our laboratory. Site-directed mutagenesis was employed to generate mutant versions of the PRRSV nsp11 construct, specifically the C112A, H129A, H144A, and K173A mutations. These mutant plasmids were created using a mutagenesis kit (TakaRa, Dalian, China). All plasmids were then confirmed by Sanger sequencing to ensure accuracy. For this study, we utilized a range of commercial antibodies. The HDAC2 mouse monoclonal antibody (mAb) was obtained from Santa Cruz Biotechnology (Santa Cruz, CA, USA). Mouse anti-Flag mAb, Mouse anti-HA mAb, and Mouse anti-β-actin mAb were purchased from Sigma-Aldrich (Sigma, Northbrook, IL, USA). IRDye-conjugated secondary antibodies were sourced from Li-Cor Biosciences. Additionally, a mouse anti-PRRSV N protein mAb was generated and purified specifically for our laboratory’s use.

### 2.3. Virus Infection and Cell Transfection

Monolayers of PAMs cells were infected with the PRRSV strain at a multiplicity of infection (MOI) of 0.1. Following incubation for 1 h at 37 °C, the virus mixture was discarded, and the cells were subsequently cultured in complete medium for an additional 24 h or specified time points. Cells were transfected with the indicated plasmids using the X-tremeGENE transfection reagent, adhering strictly to the manufacturer’s instructions (Roche, Indianapolis, IN, USA). Alternatively, cells were transduced with lentiviruses expressing HDAC2 as reported previously [30]. 

### 2.4. RNA Interference Assay

Specific small interfering (si) RNAs targeting the porcine *HDAC2* gene (si-HDAC2) and a negative control siRNA (siControl) were designed and synthesized by Sigma. Subsequently, the cells were transfected with either the si-HDAC2 or the siControl duplex, both at a concentration of 60 nM, for 24 h. This transfection was performed using the Lipofectamine RNAiMAX reagent (Invitrogen, Carlsbad, CA, USA), following the manufacturer’s instructions precisely.

### 2.5. Western Blotting Assay

Western blotting analysis was conducted as previously described with minor modifications [31]. The treated samples were harvested using radioimmunoprecipitation assay (RIPA) buffer (HaiGene, Harbin, China) supplemented with a protease inhibitor cocktail and phosphatase inhibitors (Roche, Indianapolis, IN, USA). The samples were then separated by SDS-PAGE under reducing conditions. Subsequently, the proteins were transferred onto a polyvinylidene difluoride (PVDF) membrane (Merck Millipore, Temecula, CA, USA). The membrane was blocked in 5 % (*w*/*v*) skim milk for 1.5 h and incubated with a primary antibody and subsequently treated with an appropriate IRDye-conjugated secondary antibody (Li-Cor Biosciences, Lincoln, NE, USA) for 1 h at room temperature. Finally, the membranes were scanned using an Odyssey instrument (LiCor Bio-Sciences, Lincoln, NE, USA) according to the manufacturer’s instructions.

### 2.6. Quantitative Reverse Transcription-PCR (RT-qPCR)

RT-qPCR analysis was carried out as described previously [30]. Total RNA was extracted at the designated time points postinfection or posttransfection using TRIzol reagent (TaKaRa). This extracted RNA was then used for RT-qPCR, employing specific primers listed in Table 1. Relative gene expression levels were determined using the comparative cycle threshold (∆∆CT) method [32].

### 2.7. TCID_50_ Assay

TCID_50_ assays were conducted according to the Reed–Muench method, as previously described [32]. Marc-145 cell monolayers were seeded in a 96-well plate one night prior to infection. Subsequently, the cells were infected with serial dilutions of PRRSV and incubated for 4–5 days. Daily, the cells were observed for the presence of cytopathic effect.

### 2.8. CCK-8 Assay

To assess the cytotoxicity effect of HDAC2 overexpression, a CCK-8 assay kit (Dojindo, Kumamoto, Japan) was utilized. Initially, immortalized PAM monolayers were seeded in 96-well plates and infected with the lentivirus. After 24 h of infection, CCK-8 solution was added to each well and the plate was incubated for an additional 3 h at 37 °C. Subsequently, the absorbance of the microplate was measured at 450 nm using a spectrophotometer or a microplate reader. 

### 2.9. Statistical Analysis

Analysis of GraphPad Prism 9.5.1 (GraphPad Software, Inc., La Jolla, CA, USA) was used for statistical analyses. The data are expressed as the mean ± SD. A *p* value < 0.05 was considered significant.

## 3. Results

### 3.1. PRRSV Infection Downregulates HDAC2 Expression

**Figure 1:**
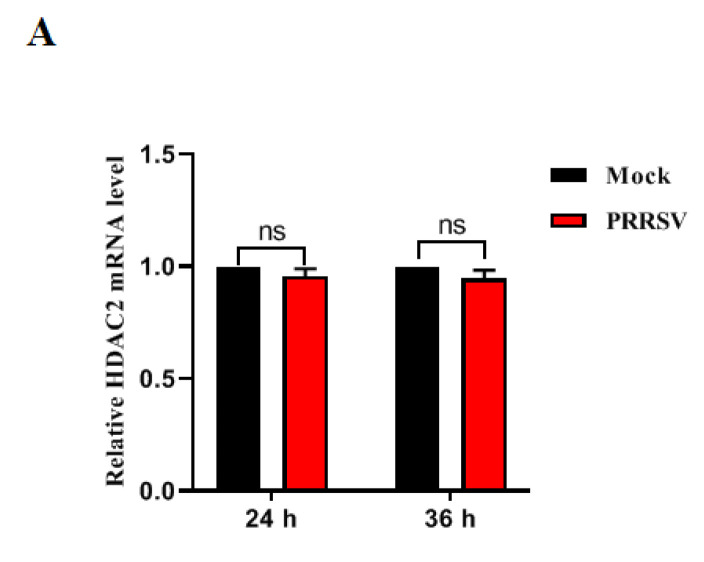

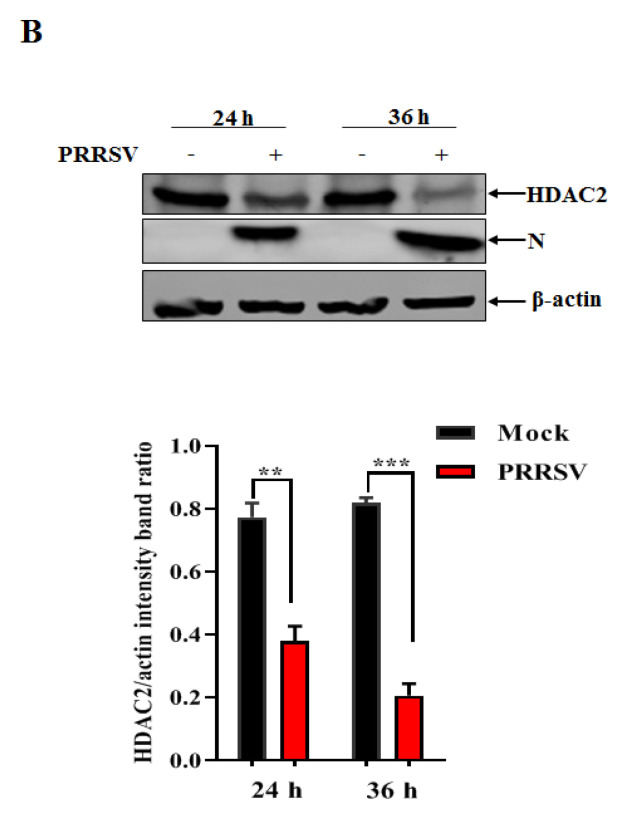
PRRSV infection reduces the HDAC2 endogenous expression. (**A**,**B**) PRRSV infection leads to the downregulation of HDAC2. PAMs were infected with PRRSV at an MOI of 0.1, and samples were collected at specific time points. The levels of HDAC2 mRNA and protein were determined by RT-qPCR (**A**) and Western blotting (**B**). The results represent three independent experiments (the means ± SD). ns means no significance **, *p* < 0.01, ***, *p* < 0.001. The *p* value was calculated using Student’s *t*-tests.

To investigate the impact of PRRSV infection on HDAC2 expression in PAMs cells, we analyzed both mRNA and protein levels of HDAC2 in PRRSV-infected PAMs. RT-qPCR results demonstrated that PRRSV infection did not significantly alter the mRNA abundance of HDAC2 at various time points (Figure 1A). However, Western blotting analysis revealed a reduction in the protein level of HDAC2 in PAMs cells infected with PRRSV compared to uninfected cells (Figure 1B). These data indicated that PRRSV infection decreases levels of HDAC2 protein in the infected cells.

### 3.2. HDAC2 Negatively Regulates PRRSV Replication

**Figure 2:**
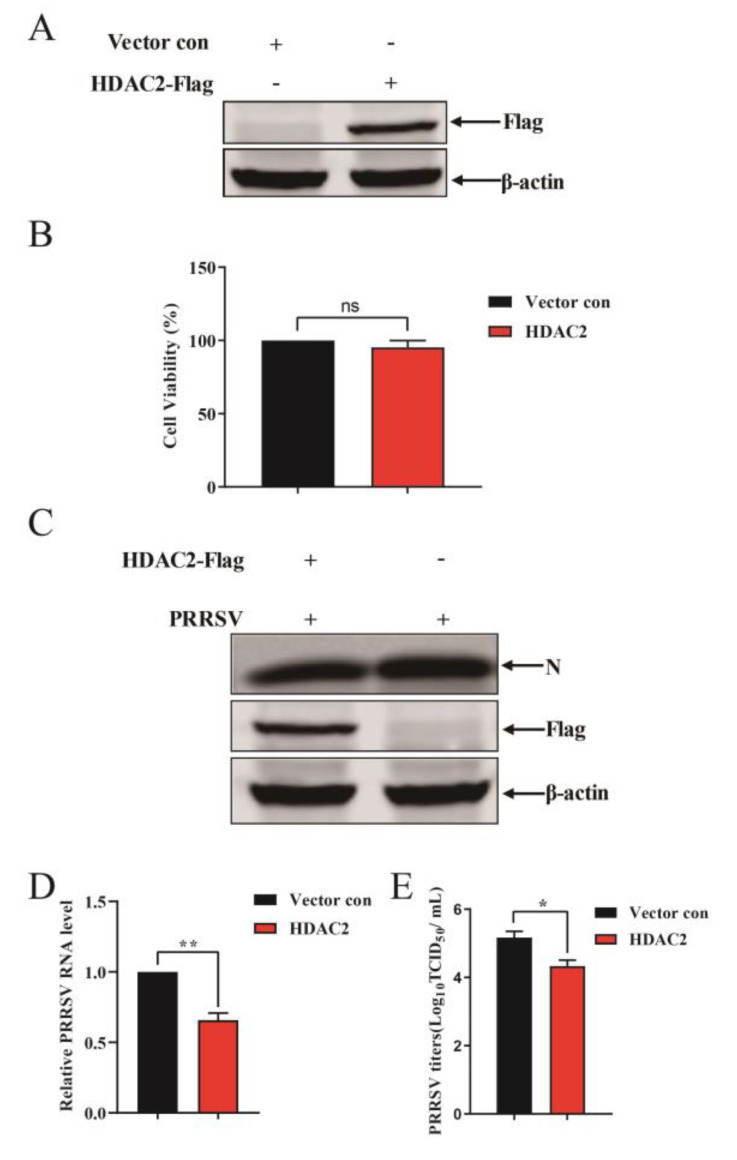
HDAC2 negatively regulates PRRSV replication. (**A**) Western blot analysis of HDAC2 expression in immortalized PAMs cells transduced with a lentiviral vector expressing HDAC2. Immortalized PAMs cells were transduced with a bicistronic lentivirus vector designed to express HDAC2. Subsequently, Western blot analysis was conducted on lysates derived from PAMs cells that had been transduced with either the HDAC2 construct or the vector control. (**B**) CCK8 assay assessed the proliferation ability of HDAC2-overexpressed PAMs. (**C**) For PAMs cells that expressed exogenous HDAC2 and were infected with PRRSV, viral N expression was evaluated by Western blotting at 24 h post-infection. (**D**) The level of viral RNA was quantified by RT-qPCR as described in panel C. (**E**) Cells were subjected to PRRSV infection for 24 h following HDAC2 overexpression for 24 h. PRRSV viral titer was calculated by TCID_50_. The results represent three independent experiments (the means ± SD). ns means no significance. * *p* < 0.05, **, *p* < 0.01. The *p* value was calculated using Student’s *t*-tests.

To investigate HDAC2′s role in PRRSV replication, we employed a lentivirus system to overexpress HDAC2 in immortalized PAMs. Immortalized PAMs cells were transduced with a bicistronic lentiviral vector expressing porcine HDAC2. After 24 h of transduction, the cells were infected with PRRSV. Western blot analysis confirmed the expression level of HDAC2 (Figure 2A). CCK-8 assay results indicated that HDAC2 overexpression did not affect the proliferation of immortalized PAMs (Figure 2B). Following PRRSV infection, immunoblotting analysis using PRRSV N antibody revealed that HDAC2 expression in PAMs cells suppresses PRRSV infectivity compared to vector control-treated cells (Figure 2C). Consistent with this, RT-qPCR results demonstrated a decrease in viral RNA levels in PAMs cells overexpressing HDAC2 (Figure 2D). Furthermore, HDAC2 overexpression led to a reduction in PRRSV progeny virus production relative to mock cells, as determined by TCID_50_ assay (Figure 2E). These data suggest that ectopic expression of HDAC2 restricts PRRSV infection in PAMs cells.

### 3.3. Knockdown of Endogenous HDAC2 Promotes PRRSV Infection

**Figure 3:**
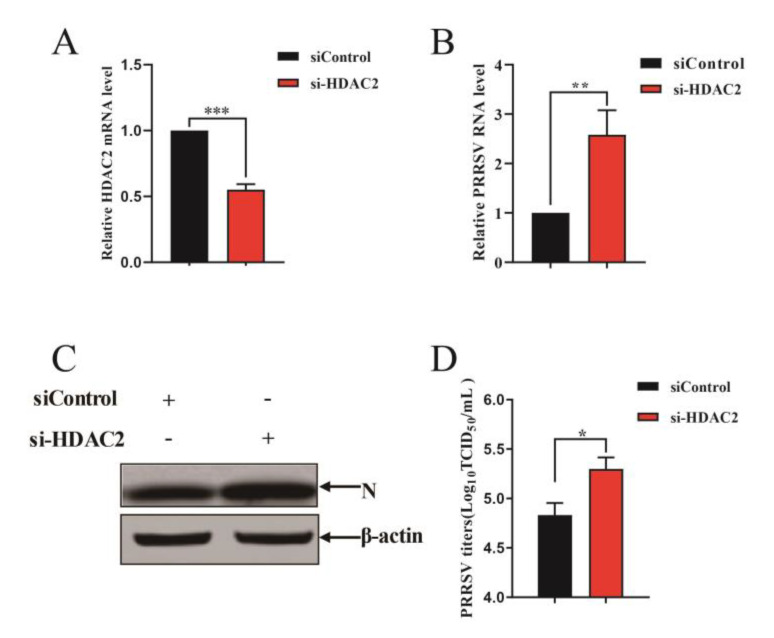
HDAC2 knockdown promotes PRRSV propagation. (**A**) Validation of HDAC2 knockdown using si-HDAC2. PAMs were transfected with either si-HDAC2 or siControl for 24 h. Total RNA was then extracted from the cell samples, and RT-qPCR was performed to assess the knockdown efficiency of si-HDAC2. (**B**–**D**) HDAC2 knockdown enhances PRRSV infection. PAMs cells were transfected with si-HDAC2 or siControl for 24 h, followed by infection with PRRSV for another 24 h. (**B**) The effect of HDAC2 knockdown on PRRSV replication was evaluated by RT-qPCR. (**C**) Western blot analysis was performed to assess the protein level of the PRRSV N protein. (**D**) To quantify PRRSV virion production, PAMs cells transfected with si-HDAC2 or siControl were infected with PRRSV, and the viral titer was determined by TCID_50_ assay. The results represent three independent experiments (the means ± SD). *, *p* < 0.05, **, *p* < 0.01, ***, *p* < 0.001. The *p* value was calculated using Student’s *t*-tests.

Next, we further characterized the antiviral activity of HDAC2 against PRRSV. We knocked down endogenous HDAC2 expression in PAMs cells using specific small interfering (si) RNAs (si-HDAC2). Negative control siRNA (siControl) was used as a reference. After 24 h of transfection, RT-qPCR analysis revealed a significant reduction in HDAC2 expression in cells transfected with si-HDAC2 compared to those transfected with siControl (Figure 3A). This validated the effectiveness of si-HDAC2 in knocking down HDAC2 expression and allowed us to use it in subsequent experiments. Next, PAMs cells were infected with PRRSV 24 h after siRNA transfection, and the infection was allowed to proceed for an additional 24 h. RT-qPCR analysis showed that knockdown of HDAC2 in PAMs cells resulted in a significant increase in PRRSV RNA levels compared to control cells (Figure 3B). Consistent with this finding, Western blot analysis demonstrated an increase in the protein level of the PRRSV N protein in cells transfected with si-HDAC2 (Figure 3C). Finally, we quantified the PRRSV virion production in PAMs cells following HDAC2 knockdown. Using the TCID_50_ assay, we found that cells transfected with si-HDAC2 produced significantly higher titers of PRRSV compared to cells transfected with the negative control siRNA (Figure 3D). All in all, these results provide further evidence for the antiviral function of HDAC2 against PRRSV infection. By both overexpressing HDAC2 and knocking it down, we have validated its role in restricting PRRSV replication in PAMs cells. 

### 3.4. HDAC2 Positively Regulates IFN Antiviral Responses

**Figure 4:**
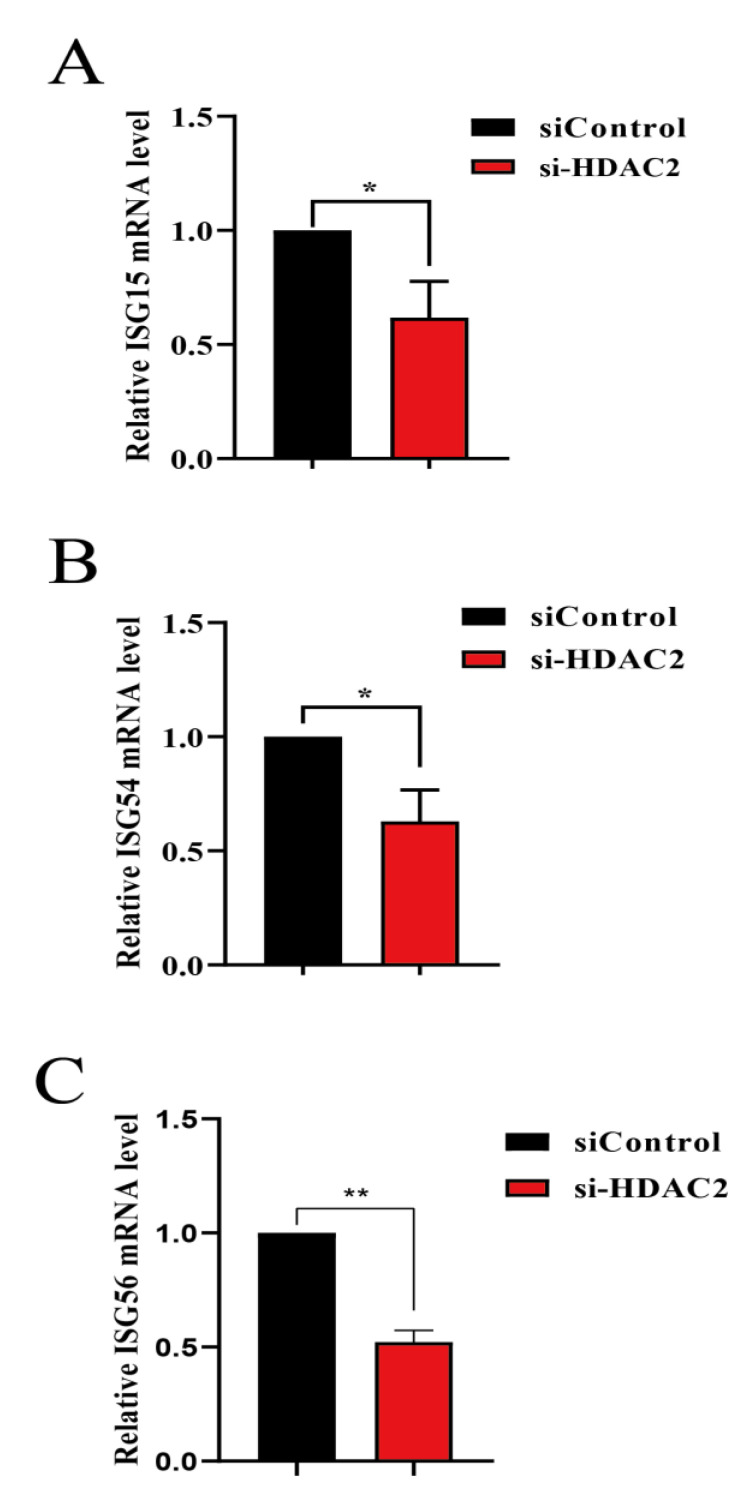
HDAC2 induced upregulated ISG expression. (**A**–**C**) PAMs cells were transfected with either si-HDAC2 or siControl for 24 h. Subsequently, the cells were treated with IFN-α for an additional 12 h to stimulate the antiviral response. After the treatment, RNA was extracted from the harvested cells for further analysis. The transcription of antiviral genes, ISG15 (**A**), ISG54 (**B**), and ISG56 (**C**), were determined by RT-qPCR using the primers in Table 1. The results represent three independent experiments (the means ± SD). *, *p* < 0.05, **, *p* < 0.01. The *p* value was calculated using Student’s *t*-tests.

Given the fact that HDAC2 can decrease PRRSV infection, it is crucial to delve deeper into the mechanisms by which HDAC2 hinders PRRSV infection. To this end, we conducted experiments to assess the role of HDAC2 in regulating IFN-induced antiviral responses. When target cells were transfected with si-HDAC2 prior to exposure to IFN-α, we measured the expression of several crucial antiviral molecules using RT-qPCR. These molecules included ISG15, ISG54, and ISG56, which are known to play important roles in the antiviral response. The results showed that the mRNA abundance of these antiviral molecules was downregulated in cells transfected with si-HDAC2 compared to control cells (Figure 4A–C). These findings suggest that HDAC2 may play a critical role in enhancing the expression of antiviral genes, thereby bolstering the cell’s defense against PRRSV infection. 

### 3.5. PRRSV nsp11 Inhibits HDAC2 Expression

**Figure 5:**
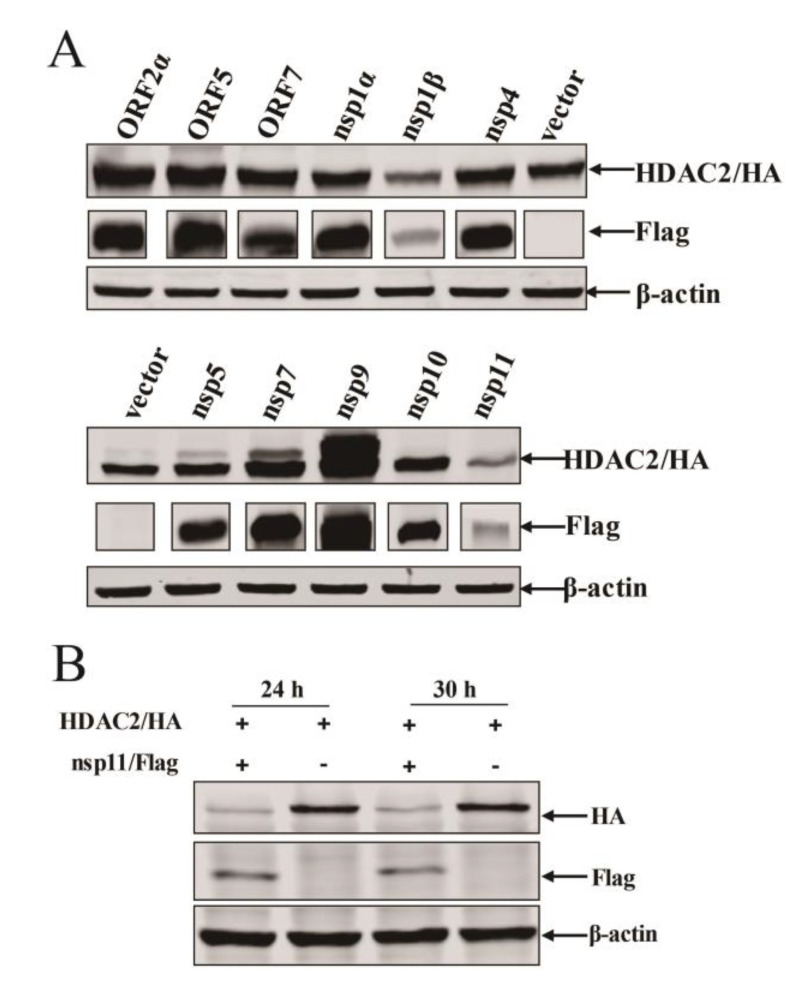
The expression of HDAC2 was inhibited by PRRSV nsp11. (**A**) HEK293T cells were co-transfected with plasmids containing gene-encoding HDAC2 and plasmids containing individual PRRSV-encoded protein. At 24 h post-transfection, cells were lysed, and immunoblots were performed to analyze protein expression. (**B**) Marc-145 cells were co-transfected with HDAC2 expression plasmid along with Flag-nsp11 plasmid followed by assessment of HDAC2 expression by Western blotting.

To unravel which viral protein is involved in the reduction of HDAC2, we performed the co-transfection assay to screen viral protein. We transfected HEK293T cells with plasmids containing gene-encoding HDAC2 and plasmids containing selected PRRSV-encoded protein. We found that some viral proteins contribute to the reduction of HDAC2 expressions to varying extents, e.g., nsp1β and nsp11, among which nsp11 could obviously inhibit the expression of HDAC2 compared with other viral proteins (Figure 5A). Therefore, in the next portion of our study, we focused on PRRSV nsp11 to investigate its role in modulating HDAC2 expressions.

To further verify the nsp11-mediated HDAC2 expression reduction, we co-transfected Marc-145 cells by HDAC2 expression plasmid along with Flag-nsp11 plasmid. We also examined HDAC2 expression by Western blotting analysis. Consistently, HDAC2 protein abundance decreased in PRRSV nsp11 transfected cells (Figure 5B). Altogether, the obtained findings show that PRRSV nsp11 inhibits HDAC2 protein expression, suggesting a mechanism by which the virus antagonizes the host’s innate immune response. 

### 3.6. nsp11 Mediated HDAC2 Reduction in an Endonuclease Activity Dependent Manner

**Figure 6:**
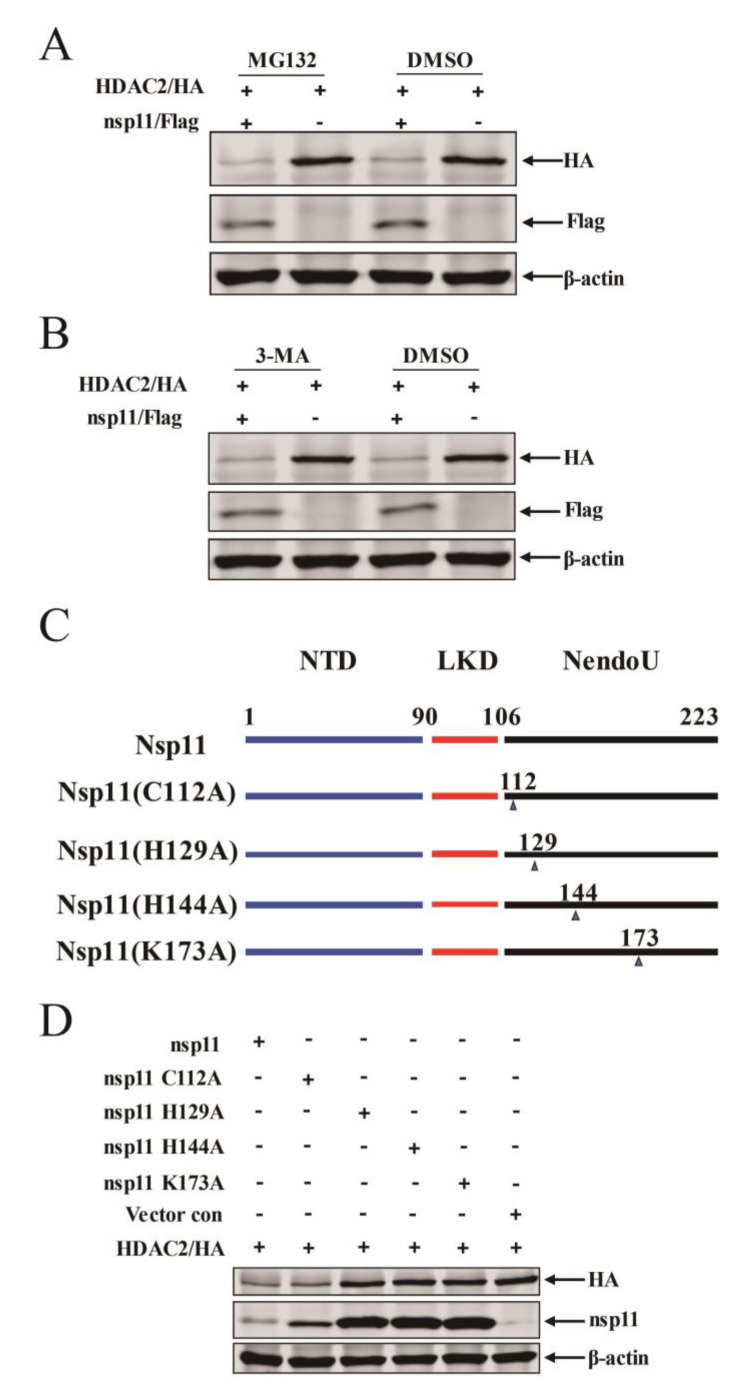
H^129^, H^144^, and K^173^ are necessary for nsp11-mediated HDAC2 reduction. (**A**) HDAC2 expression plasmid and Flag-nsp11 plasmid were transfected into HEK293T cells, and after 18 h of incubation, the cells were treated with either MG132 or DMSO for an additional 6 h. Subsequently, the cells were lysed, and immunoblots were performed using anti-Flag and anti-HA antibodies to assess the expression levels of nsp11 and HDAC2, respectively. (**B**) HEK293T cells were co-transfected with HDAC2 expression plasmid and Flag-nsp11 plasmid. Following transfection, the cells were treated with 3-MA, an autophagy inhibitor, for further culture. After the treatment, the cells were lysed, and immunoblots were performed to analyze the expression levels of nsp11 and HDAC2. (**C**) Schematic representation of nsp11 and its individual mutants (C112A, H129A, H144A, or K173A). These mutants were generated by mutating specific amino acid residues in nsp11 to alanine. (**D**) In HEK293T cells subjected to HDAC2 expression plasmid together with Flag-nsp11 plasmid or constructed mutant plasmids co-transfection, after 24 h, cells were lysed and analyzed by immunoblots.

To gain a deeper understanding of the mechanism underlying nsp11-mediated HDAC2 expression reduction, we examined the predominant degradation pathway responsible for HDAC2 degradation. HEK293T cells were co-transfected with plasmids encoding HDAC2 and Flag-tagged nsp11, followed by treatment with either the protease inhibitor MG132 or the autophagy inhibitor 3-methyladenine (3-MA). Western blotting was performed to assess the amount of HDAC2 protein expression. As shown in Figure 6A, treatment with MG132 did not restore the HDAC2 protein level in nsp11-transfected cells, suggesting that HDAC2 degradation is not mediated by the proteasome-dependent pathway. Similarly, treatment with 3-MA also failed to rescue HDAC2 expression (Figure 6B), indicating that autophagy is not involved in this process.

It has been reported that His129, His144, Lys173, and Cys112 are critical amino acid residues for the endonuclease activity of PRRSV nsp11 and play a pivotal role in nidovirus replication and pathogenesis [17], We hypothesized that these residues might also be involved in nsp11-mediated HDAC2 reduction. To test this hypothesis, we generated Flag-tagged mutant versions of nsp11 by mutating each of these residues to alanine (C112A, H129A, H144A, and K173A) (Figure 6C). HEK293T cells were then co-transfected with plasmids encoding HDAC2 and either the wild-type nsp11 or the mutant versions. Western blotting was used to assess HDAC2 expression. Our results demonstrated that overexpression of the C112A mutant did not significantly affect HDAC2 expression compared to the wild-type nsp11. However, overexpression of the H129A, H144A, and K173A mutants significantly blocked the reduction of HDAC2 expression induced by nsp11 (Figure 6D). These findings suggest that the endonuclease activity of nsp11, specifically mediated by the His129, His144, and Lys173 residues, is critical for its ability to reduce HDAC2 expression. 

### 3.7. PRRSV nsp11 Antagonizes the Antiviral Response of HDAC2 in an Endonuclease Activity Dependent Manner

**Figure 7:**
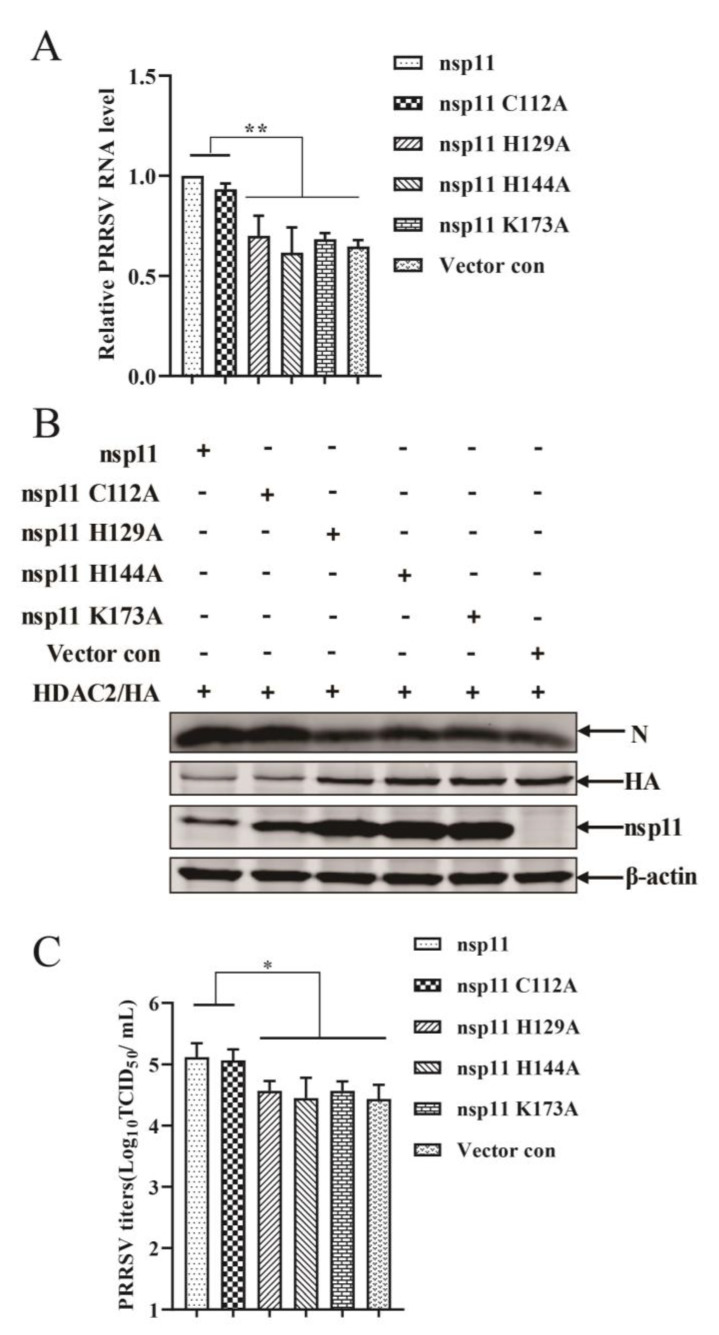
Nsp11 antagonizes host innate immune response of HDAC2 dependent on its endoribonuclease activity. (**A**–**C**) HDAC2 and either nsp11 or its mutant versions (C112A, H129A, H144A, or K173A) were transfected into Marc-145 cells as indicated. Subsequently, the cells were infected with PRRSV. After incubation, the Marc-145 cells were harvested to assess the levels of viral PRRSV N mRNA (**A**), N protein expression (**B**), and virus titers (**C**). The results represent three independent experiments (the means ± SD). *, *p* < 0.05, **, *p* < 0.01. The *p* value was calculated using Student’s *t*-tests.

To further investigate whether the antiviral response of HDAC2 is antagonized by PRRSV nsp11 in a manner dependent on its endonuclease activity, HDAC2 expression plasmid and Flag-nsp11 plasmid or nsp11 mutant plasmids were co-transfected into Marc-145 cells for 24 h. Following transfection, the cells were infected with PRRSV and incubated for 24 h. Firstly, the levels of viral RNA were significantly increased in cells transfected with either the wild-type nsp11 or the C112A mutant compared to cells transfected with the mutants lacking the endonuclease activity-critical residues H^129^, H^144^, or K^173^ (Figure 7A). Secondly, the expression of the PRRSV N protein was also significantly upregulated in cells transfected with wild-type nsp11 or the C112A mutant compared to the other mutant-transfected cells (Figure 7B). Lastly, we measured the virus titers in the transfected and infected cells. Consistent with the previous findings, the virus titers were significantly higher in cells transfected with wild-type nsp11 or the C112A mutant compared to cells transfected with the endonuclease activity-deficient mutants (Figure 7C). Taken together, these results suggest that PRRSV nsp11 antagonizes the antiviral response of HDAC2 in an endonuclease activity-dependent manner, thereby benefiting PRRSV replication. 

## 4. Discussion

Intrinsic immunity serves as the initial line of defense in the host, effectively limiting the spread of viruses and orchestrating the subsequent adaptive immune responses. Viral pathogen-associated molecular patterns (PAMPs), encompassing viral RNAs and intermediate RNAs, are promptly recognized by host–pathogen recognition receptors (PRRs). This recognition subsequently initiates intracellular sensing mechanisms, involving key players like the interferon (IFN) regulatory factor (IRF) family members and the nuclear factor kappa-light-chain-enhancer of activated B cells (NF-κB) [33]. These transcription factors induce the expression of type I interferon (IFN-I), and, subsequently, secreted IFN-I binds to the IFN receptor, activating the downstream expression of IFN-stimulated genes (ISGs) [34,35,36]. However, viruses must counter IFN’s powerful responses by regulating or evading these defenses to facilitate viral infection [37]. Notably, previous studies revealed that PRRSV is susceptible to the antiviral acticity of IFNs both in vivo and in vitro [38]. Therefore, PRRSV, like other viruses, has evolved plenty of strategies against initial antiviral host responses [15,39,40]. 

It has been previously reported that PRRSV infection interferes with the MAVS activation in the RIG-I signaling pathway, thereby inhibiting the production of IFN-β [41]. PRRSV nsp1β significantly blocks dsRNA-induced phosphorylation and nuclear translocation of IRF3, a key transcription factor involved in IFN-I expression [15]. Additionally, the 3C-like protease of PRRSV and equine arteritis virus (EAV) disrupt type I IFN signaling by cleaving NEMO, a critical adaptor protein for NF-κB activation [17]. Altogether, PRRSV-encoded protein could limit host antiviral restriction factors to establish a persistent infection. In the present study, we showed that the HDAC2 protein level was decreased during PRRSV infection. Furthermore, through overexpression and knockout experiments, we demonstrated that HDAC2 could significantly inhibit PRRSV replication. Taking the results together, we evidenced that HDAC2 was verified as the host restriction factor that limits PRRSV replication. Further analysis revealed that nsp11 is crucial in PRRSV-regulated HDAC2 expression. Specifically, nsp11 antagonizes the antiviral response of HDAC2 in a manner dependent on its endonuclease activity, thereby revealing a novel antagonistic mechanism employed by PRRSV to counteract the host’s antiviral defenses.

PRRSV nsp11 is a well-established multifunctional protein known for its endoribonuclease and deubiquitinating activities [42,43], and it plays a crucial role in virus replication, making it a crucial component in the PRRSV lifecycle [44,45]. Further studies have displayed that nsp11 functions as a potent antagonist of the IFN response, employing various mechanisms to suppress IFN production. For instance, nsp11 has been shown to suppress the activation of transcription factors IRF3 and NF-κB, thereby inhibiting the production of type I IFNs [46,47]. Sun et al. reported that nsp11 reduces the levels of transcripts and proteins related to MAVS, RIG-I, and ISG15 [46,48]. Additionally, nsp11 is capable of inhibiting NF-κB activation by removing ubiquitin chains from IκBα [49]. It also enhances its ability to suppress type I IFN production by removing linear ubiquitination targeting NEMO in conjunction with OTULIN, a protein with linear linkage specificity [50], and nsp11 can adopt a autolysosome pathway for CH25H degradation [51]. Moreover, nsp11 is also reported to antagonize host innate immunity by targeting IRF9 through a mechanism independent of its endoribonuclease activity [52]. However, nsp11 can antagonize the antiviral activity of PCSK9 in a manner dependent on its endoribonuclease activity [53]. 

In summary, our study adds a new dimension to the understanding of nsp11’s antiviral antagonistic functions. We have discovered that nsp11 inhibits the antiviral activity of HDAC2 by mediating its degradation through a NendoU activity-dependent mechanism. This finding broadens our knowledge of the pathogenesis of PRRSV and underscores the complexity of the host–virus interactions during infection.

## Figures and Tables

**Table 1:** Primers used in this study.

Primer	Forward (5′→3′)	Reverse (5′→3′)
HDAC2	TTTGGTACCATGGCGTACAGTCAGGGAGGCGG	TTTCTCGAGTCAAGGGTTGCTGAGCTGTTCTGA
qHDAC2	CTTGCCATCCTTGAGTTA	TTTAGCGTGACCTTTGAC
qPRRSV-ORF7	AGATCATCGCCCAACAAAAAC	GACACAATTGCCGCTCACTA
qISG15	CCTGTTGATGGTGCAAAGCT	TGCACATAGGCTTGAGGTCA
qISG54	CATTGACCCTCTGAGGCAAG	AGCGTGTCCTATTAGTTCC
qISG56	CATACATTTCCACTATGG	TACTCCAGGGCTTCATTCA
qβ-actin	CTTCCTGGGCATGGAGTCC	GGCGCGATGATCTTGATCTTC

## Data Availability

The data that support the findings of this study are available from the corresponding author upon reasonable request.
